# Bromelain as the enzyme of the future: A global bibliometric mapping of its applications and emerging trends

**DOI:** 10.1002/jsfa.70751

**Published:** 2026-05-28

**Authors:** Genésio José da Silva Neto, Tonny Cley Campos Leite, Mônica Tejo Cavalcanti, Geany Targino Sousa Pedrosa, Rerisson do Nascimento Alves, Amanda Reges de Sena, Mônica Correia Gonçalves

**Affiliations:** ^1^ Center for Agrifood Science and Technology, Federal University of Campina Grande Pombal Brazil; ^2^ Department of Antibiotics Federal University of Pernambuco Recife Brazil; ^3^ Federal Institute of Education, Science, and Technology of Pernambuco Barreiros Brazil; ^4^ Center for Biological and Health Sciences, Federal University of Campina Grande Campina Grande Brazil; ^5^ Center for Bioeconomy Medical Biotechnology and Innovation in the Caatinga Campina Grande Brazil; ^6^ Center for Science and Technology, Federal University of Campina Grande Campina Grande Brazil

**Keywords:** bibliometrics, Bromeliaceaea, plant protease, technological applications, trends, VOSviewer

## Abstract

**INTRODUCTION:**

Bromelain, a complex mixture of proteolytic enzymes primarily extracted from plants of the Bromeliaceae family, has gained prominence because of its broad industrial applicability, particularly in the food, pharmaceutical and cosmetic sectors. Despite its wide use, a systematic understanding of recent scientific advances remains limited, highlighting the importance of bibliometric approaches.

**METHODS:**

A bibliometric analysis was conducted using publications indexed between 2020 and 2024, retrieved from scientific databases, and analyzed through quantitative indicators of scientific production, collaboration networks and research trends.

**RESULTS:**

The analysis demonstrated a consistent growth in scientific output, with research leadership by countries such as China, India and Italy, reflecting strong governmental investment and well‐established research frameworks. In the food industry, bromelain is applied in meat tenderization, beverage clarification and as a processing aid. Recent studies emphasize its role in the production of protein hydrolysates with bioactive properties, including antioxidant and antihypertensive activities. In the pharmaceutical field, its anti‐inflammatory, analgesic and antitumoral effects are explored in the treatment of burns and tumors, as well as its potential neuroprotective applications. In cosmetics, bromelain is used in exfoliating formulations and dentifrices for stain removal.

**CONCLUSION:**

Bromelain research extends across multiple disciplines, promoting advances in biological sciences, medicine and chemical engineering. Recent efforts have focused on identifying alternative sources beyond pineapple, aiming to obtain enzymes with improved stability and functionality under diverse processing conditions. These advances reinforce bromelain's potential as a versatile and sustainable biotechnological resource. © 2026 The Author(s). *Journal of the Science of Food and Agriculture* published by John Wiley & Sons Ltd on behalf of Society of Chemical Industry.

## INTRODUCTION

Bromelain is a collective name for a complex of several proteolytic enzymes found in plants of the Bromeliaceaea family, with the pineapple being its most prominent representative. It is a glycoprotein that possesses an oligosaccharide residue covalently linked to the polypeptide chain.^1^, ^2^

The Bromeliaceaea family occurs in all Brazilian states, with the highest concentration in the phytogeographic domains of the Amazon, Caatinga, Cerrado, Atlantic Forest, Pampa and Pantanal. Despite its wide distribution across Brazil, it is not endemic to the country and can be found in other countries across the Americas.^3^ This plant diversity contributes to the exploration of bromelain in various industrial contexts.

Species in this family are found predominantly in tropical and subtropical regions, growing under varying humidity conditions ranging from humid forests to arid areas. This adaptive success is supported by physiological and morphological mechanisms, such as crassulacean acid metabolism (CAM) photosynthesis metabolism and morphological innovations, including the ‘tank’ growth habit and the presence of leaf trichomes specialized in water storage and absorption.^4^ This ecological plasticity results in a vast biological diversity that contributes to the exploitation of bromelain in various industrial contexts.

Bromelain can be used to hydrolyze the protein complexes that cause turbidity during fermentation in beer production, thereby contributing to the stabilization process.^5^ In meat processing, it is employed for tenderization.^6^ Furthermore, there are reports of its application in producing hydrolysates used in functional foods, dietetic and dietary protein supplements, protein isolates,^7^, ^8^ and in milk coagulation for cheese production.^9^

Given the versatility and industrial impact of bromelain, it is essential to comprehend the evolution of research concerning its applications. In this context, bibliometric analysis emerges as a strategic tool to assess scientific and technical productivity through quantitative indicators derived from publications in a specific field of knowledge.^10^ This methodology allows for the identification of trends, institutional collaborations, and the current landscape of scientific and technological development related to bromelain.^11^ It can be used to analyze trends in the use of bromelain.

Therefore, the application of bibliometric methods not only enables a systematic evaluation of scientific production, but also allows the identification of research hotspots, emerging trends and knowledge gaps in the field of bromelain. These insights are essential for guiding future investigations and supporting the development of innovative and industrial applications. In this context, the present study aimed to conduct a bibliometric mapping of publications related to its applications, seeking to identify the main trends and scientific advancements during the period from 2020 to 2024.

## METHODS

The bibliometric data were collected using the Scopus database (https://www.scopus.com) and the data were collected on 20 September 2025. The search was performed using the search within ‘Article title, abstract and keywords’ field. The following terms were used for the search strategy: ‘bromelain*’ or ‘bromelain applications*’ or ‘bromelain in food industry*’ or ‘bromelain in the chemical industry*’ or ‘bromelain enzymatic*’ or ‘bromelain bioactive peptides production*’ or ‘bromelain functional properties*’ or ‘bromelain plant‐based proteases*’ or ‘industrial potential of bromelain*’ or ‘bromelain bioprospecting*’.

The bibliometric analysis considered the period from 2020 to 2024 and included only original research articles with experimental data. The following were excluded from the analysis: review articles, conference proceedings, book chapters, academic dissertations/theses and patents. The retrieved data were exported in CVS format from Scopus to ensure reproducibility and were imported directly into VOSviewer (https://www.vosviewer.com) for analysis. Data were collected by selecting all fields titled ‘Citation Information’ and ‘Bibliographical Information’, as well as ‘Abstract & Keywords’, ‘Funding Details’ and ‘Other information’.

Synonyms and variations of key terms were included using the asterisk () operator to capture all relevant publications*. To compile the ranking of the top 10 articles, the number of publications within each category was adopted as the criterion. These categories included: journals, affiliated institutions, funding agencies, countries, articles and areas of study.

The collaboration networks among authors, journals, institutions, and keywords were generated using the VOSviewer, version 1.6.20, following the methodological procedures proposed by Eck and Waltman^12^, ^13^ and adapted by Alves *et al*.^14^ The citation technique was used for analyzing authors, institutions and journals, whereas the co‐occurrence analysis was applied to the keywords. All parameters in VOSviewer, including the counting method, minimum number of occurrences for nodes and network normalization options, were maintained as default to ensure reproducibility of the results.

## RESULTS AND DISCUSSION

The analysis of scientific output over time, as illustrated in Fig. 1, reveals a continuous and consistent growth in bromelain research during the period from 2020 to 2024. Starting with 151 publications in 2020, the number of articles increased to 188 in 2021. After a slight decrease in 2022 (171 publications), which may be associated with residual impacts of the COVID‐19 pandemic on research productivity, production resumed its growth rate, reaching 177 publications in 2023, and peaking at 220 publications in 2024, likely reflecting the recovery and growing interest in bromelain applications across industrial and biotechnological sectors.

**Figure 1:**
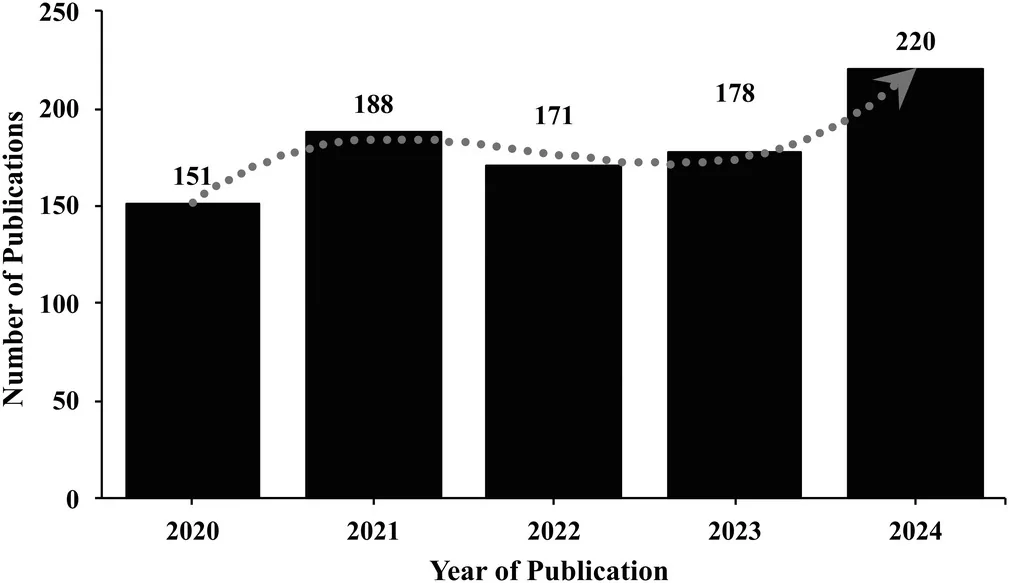
Annual distribution of publications related to the application of bromelain in the database of Scopus (2020–2024). Data collected on 20 September 2025.

In addition to the bibliometric indicators, representative studies were selected throughout this section to illustrate the main applications and research trends of bromelain. These articles were chosen as illustrative examples rather than as an exhaustive selection.

In 2020, Santos *et al*.^15^ evaluated the use of dehydrated pineapple by‐products, enriched with bromelain via hydrostatic pressure treatment, aiming to improve the tenderization and other properties of beef. Tougher steaks from the silverside cut were marinated for different times and bromelain concentrations and analyzed for pH, color, marinade yield, hardness and histological characteristics before and after cooking. The study demonstrated that the combination of longer marination times (12–24 h) and higher bromelain concentrations resulted in lower meat hardness, higher marinade yield, and lighter color. The results indicate that the bromelain present in pineapple by‐products can be used to add value to meat cuts of lower commercial value.

Still in 2020, Haiyan *et al*.^16^ investigated the effect of bromelain on breast cancer cells (MCF7 and MDA‐MB‐231) as a safe therapeutic alternative. Different parts of the pineapple (pulp, peel, peduncle, young stem and mature stem) were used to extract the bromelain, with the enzyme extracted from the fruit's peduncle proving to be the most effective in inhibiting cell growth. The MCF7 cell line showed greater sensitivity compared to the MDA‐MB‐231 cell line. The antitumor activity involved the upregulation of p53 and Bax, the downregulation of Cox‐2 and Bcl‐2, and the possible blockade of nuclear factor‐kappa B, indicating that bromelain acts by modulating apoptotic and inflammatory pathways. The study highlights the potential of bromelain as a safe bioactive agent in the development of strategies against breast cancer.

In 2021, hydrogels were developed by copolymerization of acrylamide (AAm) with polyvinyl alcohol (PVA) or polyethylene glycol (PEG) to evaluate the incorporation and controlled release of bromelain, aiming at topical applications in the treatment of burns and inflammation. Coco *et al*.^17^ incorporated bromelain into the PVA hydrogels (Bro‐PVA‐co‐AAm) and PEG hydrogels, achieving a relative protein recovery of 91.3% and 88.9%, respectively, in addition to a relative enzymatic activity of 88.9% and 53.8%. After 24 h, less than 5% (relative release) of the incorporated protein was released, whereas enzymatic activity reached up to 60% in the PVA hydrogels and 100% in the PEG hydrogels. The analysis of the mechanical properties indicated that the PVA hydrogels demonstrated greater structural compatibility with biological conditions because of their lightweight, mucoadhesive and resilient architecture, combining softness and stability. These results demonstrate that the PVA‐co‐AAm hydrogels can function as effective controlled‐release systems for bromelain, preserving its enzymatic activity and potential for biomedical applications.

The following year, Khazaeel *et al*.^18^ evaluated the protective effects of bromelain on male reproductive function impaired by bisphenol A (BPA), an endocrine disruptor known to increase oxidative stress and testicular damage. Sixty male mice were distributed into six groups, including control, bromelain, BPA and combinations of BPA with bromelain, administered orally for 35 days. BPA significantly reduced sperm count, motility, viability and normal morphology, in addition to decreasing testosterone levels, the expression of estrogen receptors (ERα and Erβ), and the activity of antioxidants such as glutathione peroxidase, catalase and superoxide dismutase, at the same tome as increasing spermatic anomalies and malondialdehyde levels. The co‐administration of bromelain attenuated these effects, promoting a significant improvement in spermatic parameters, hormone levels and antioxidant activity, and reducing oxidative stress. The results indicate that bromelain exerts a protective effect on male reproduction, mitigating BPA‐induced damage.

In 2023, Abduh *et al*.^19^ investigated the production of protein hydrolysates from black soldier fly larvae (BSFL) (*Hermetia illucens* L.) using acid or alkaline pretreatments followed by bromelain hydrolysis. Protein extraction with hydrochloric acid and sodium hydroxide was performed at different temperatures (25–50 °C) and times (30–60 min), achieving a maximum protein recovery of 38.42% with sodium hydroxide at 50 °C for 30 min. The hydrolysate obtained using bromelain was optimized in terms of enzyme concentration (30 g kg^−1^), pH (8) and time (24 h), resulting in a degree of hydrolysis of 58.52% and antioxidant activity with a half maximal inhibitory concentration of 1.15%. Amino acid analysis revealed a predominance of glutamic acid (37 g kg^−1^), leucine (21 g kg^−1^) and valine (20 g kg^−1^). This study demonstrates the potential of bromelain in generating bioactive protein hydrolysates from BSFL.

In 2024, Lia *et al*.^20^ compared thermal and enzymatic methods for producing fish collagen hydrolysates, evaluating their characteristics and anti‐aging potential. Thermal hydrolysis was performed at different temperatures (50–100 °C) and times (1–5 h), whereas enzymatic hydrolysis utilized bromelain (10 U mL^−1^) at 50 °C for 1 h. The hydrolysates were characterized by electrophoresis, Lowry assay, hydroxyproline content, scanning electron microscopy, Fourier transform infrared spectroscopy and differential scanning calorimetry. The results showed that thermal hydrolysis produced denatured protein, whereas enzymatic hydrolysis with bromelain generated low molecular weight peptides with a degree of hydrolysis of 60%, better preserving the structural and physical characteristics of the collagen. The findings indicate that bromelain is efficient for producing peptidic collagen capable of penetrating the skin, thereby enhancing its potential anti‐aging effect.

The list of the top 10 most productive authors, ranked by the number of publications (*n*) and their percentage representation (%) of the total analyzed documents, according to the Scopus database, is found in Table 1. The author Morris, D. L. stands out as being the most productive, with 17 publications, which corresponds to 1.88% of the total analyzed documents (904). Subsequently, Holyavka, M.G. and Artyukhov, V.G. follow as the second and third most productive authors, with 15 and 14 publications, respectively.

**Table 1:** Ranking of authors with the highest number of publications in research on bromelain application, according to bibliometric analysis in the Scopus database (2020–2024)

Ranking	Name	*n*	%
1	Morris, D. L.	17	1.88
2	Holyavka, M.G.	15	1.66
3	Artyukhov, V.G.	14	1.55
4	Shoham, Y.	12	1.33
5	Valle, S.J.	10	1.11
6	Pillai, K.	10	1.11
7	Mekkawy, A.H.	10	1.11
8	Akhter, J.	9	1.00
9	Sorokin, A.V.	9	1.00
10	Mudgil, P.	9	1.00

*Note: n*, number of publications. Source: data collected on 20 September 2025.

One of the most relevant studies from the research group of Morris, D. L. was the evaluation of the effect of BromAc® (Mucpharm Pty Ltd, Kogarah, NSW, Australia), a combination of bromelain (a proteolytic pineapple enzyme) and acetylcysteine (a mucolytic agent), on the dissolution of mucus plugs in an *ex vivo* ovine pulmonary model. BromAc® significantly reduced airway resistance and mucin viscosity, surpassing the effects of the isolated agents, without affecting compliance or tidal volume, demonstrating its efficacy as a mucolytic agent.^21^

The number of publications per individual author is relatively low in relation to the total volume of data, indicating that scientific production is not concentrated in a few authors but is rather distributed among a larger number of researchers. The presence of multiple authors with the same number of publications suggests the existence of research groups or collaborators with similar productivity. Although the ranking identifies the main contributors, the low percentage of their publications relative to the total corpus suggests that the research area in question is vast, with the collaboration and contribution of a broad and diverse scientific community.

The chart, generated by VOSviewer (Fig. 2), represents the collaboration network among the main authors. The network shows that, although the most cited authors are connected, the collaboration pattern is still relatively limited. Some authors, such as Muthayya, P., Buehler, P. K., and Steiger, P., present higher total link strength, suggesting a more active role in connecting different research groups. On the other hand, authors with a high number of citations, such as Hoeksma, H. and Korzeniowski, T., exhibit lower connectivity, indicating that their contributions are more isolated within the network. Overall, the structure suggests that bromelain research is developing with increasing collaboration, but still lacks strong integration among research groups.

**Figure 2:**
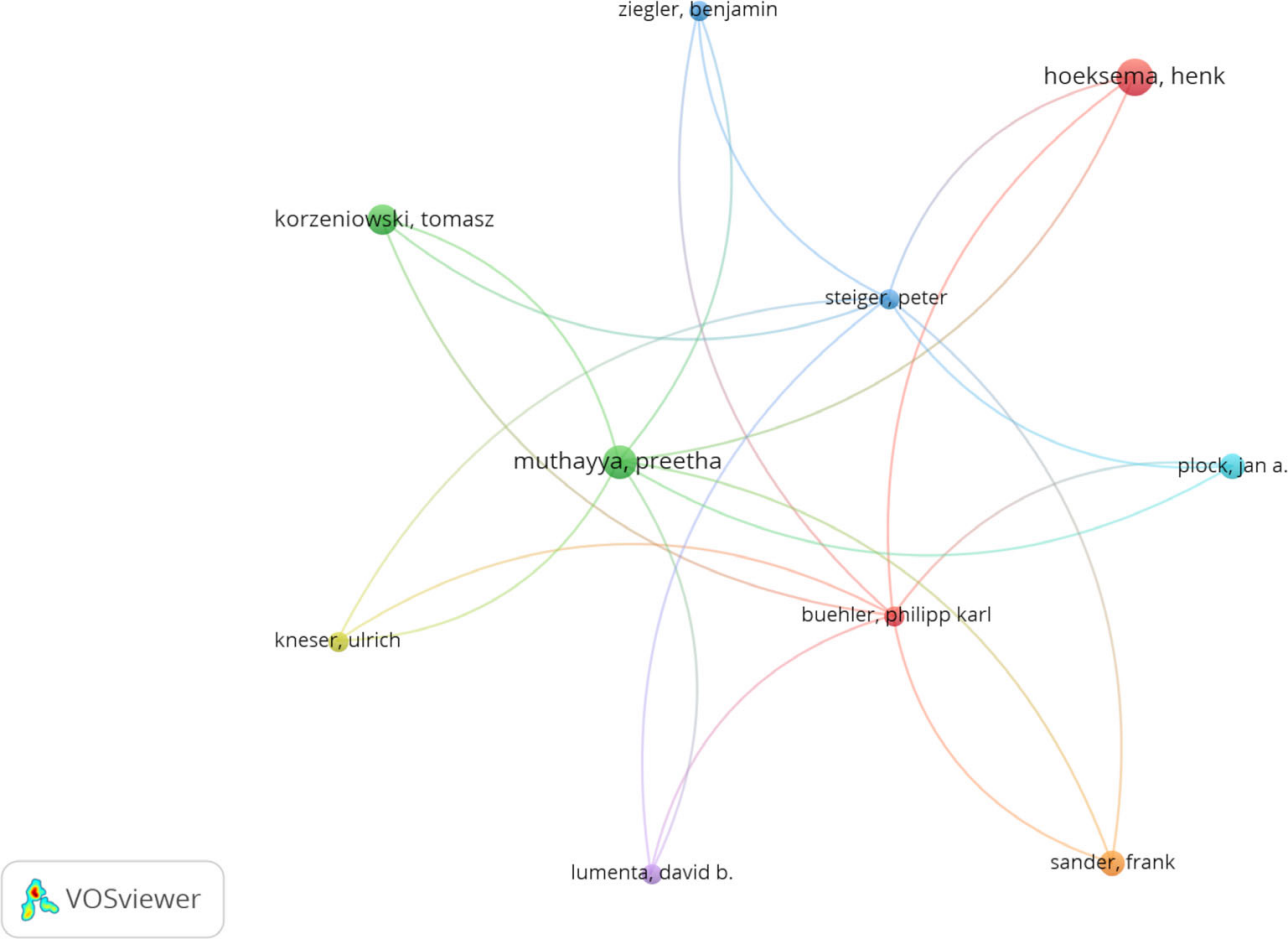
Visualization map for the 10 most cited authors in bromelain application research, according to bibliometric analysis in the database Scopus (2020–2024). Data collected on 20 September 2025. Analysis performed using VOSviewer, version 1.6.20.

The ranking of the top 10 journals with the highest number of publications, according to the Scopus database, is found in Table 2. The journal *Foods* stands out in the first position with 20 publications, followed by *Molecules* and *Burns*, with 18 and 17, respectively. These data suggest that these journals serve as the main platforms for the dissemination of knowledge in this field, according to the Scopus database, which may reflect their relevance and visibility within the scientific community.

**Table 2:** Ranking of journal authors with the highest number in bromelain application research, according to bibliometric analysis in the Scopus database (2020–2024)

Ranking	Source title	*n*	%	IF	CS	Journal quartile
1	*Foods*	20	2.21	5.10	8.70	Q1
2	*Molecules*	18	1.99	4.60	8.60	Q1
3	*Burns*	17	1.88	4.70	2.90	Q1
4	*International Journal of Biological Macromolecules*	14	1.55	10.30	8.50	Q1
5	*Food Chemistry*	14	1.55	9.80	18.30	Q1
6	*Journal of Clinical Medicine*	12	1.33	2.90	5.20	Q1
7	*Scientific Reports*	10	1.11	3.90	6.70	Q1
8	*LWT*	10	1.11	13.6	6.60	Q1
9	*Science and Technology of Food Industry*	9	1.00	0.75	1.40	Q4
10	*Applied Sciences Switzerland*	9	1.00	2.50	5.50	Q2

*Note:* IF, Impact Factor 2024; CS, CiteScore 2024. Source: data collected on 20 September 2025.

Although *Foods* is the most cited in terms of volume, the journals *LWT* and the *International Journal of Biological Macromolecules* exhibit the highest Impact Factors (13.6 and 10.3, respectively), indicating that the articles published in these journals are, on average, more influential or highly cited. Similarly, *Food Chemistry* and *Molecules* possess the highest CiteScores, at 18.3 and 8.6, respectively, suggesting that these journals are highly relevant within the Scopus citation base.

This discrepancy between the volume of citations and the impact metrics reveals that, despite a journal being a popular publishing channel, the most influential research may be dispersed across high‐quality and reputable journals. The *Foods* cluster focuses on a broader aspect of food science, such as the identification and characterization of new peptides derived from plant proteins.^22^

The journal visualization map (Fig. 3) complements the analysis in Table 2 by illustrating the collaboration network among the most relevant publishing journals. The absence of co‐citation links among the main journals suggests that articles published in one of these journals rarely cite articles published in another.

**Figure 3:**
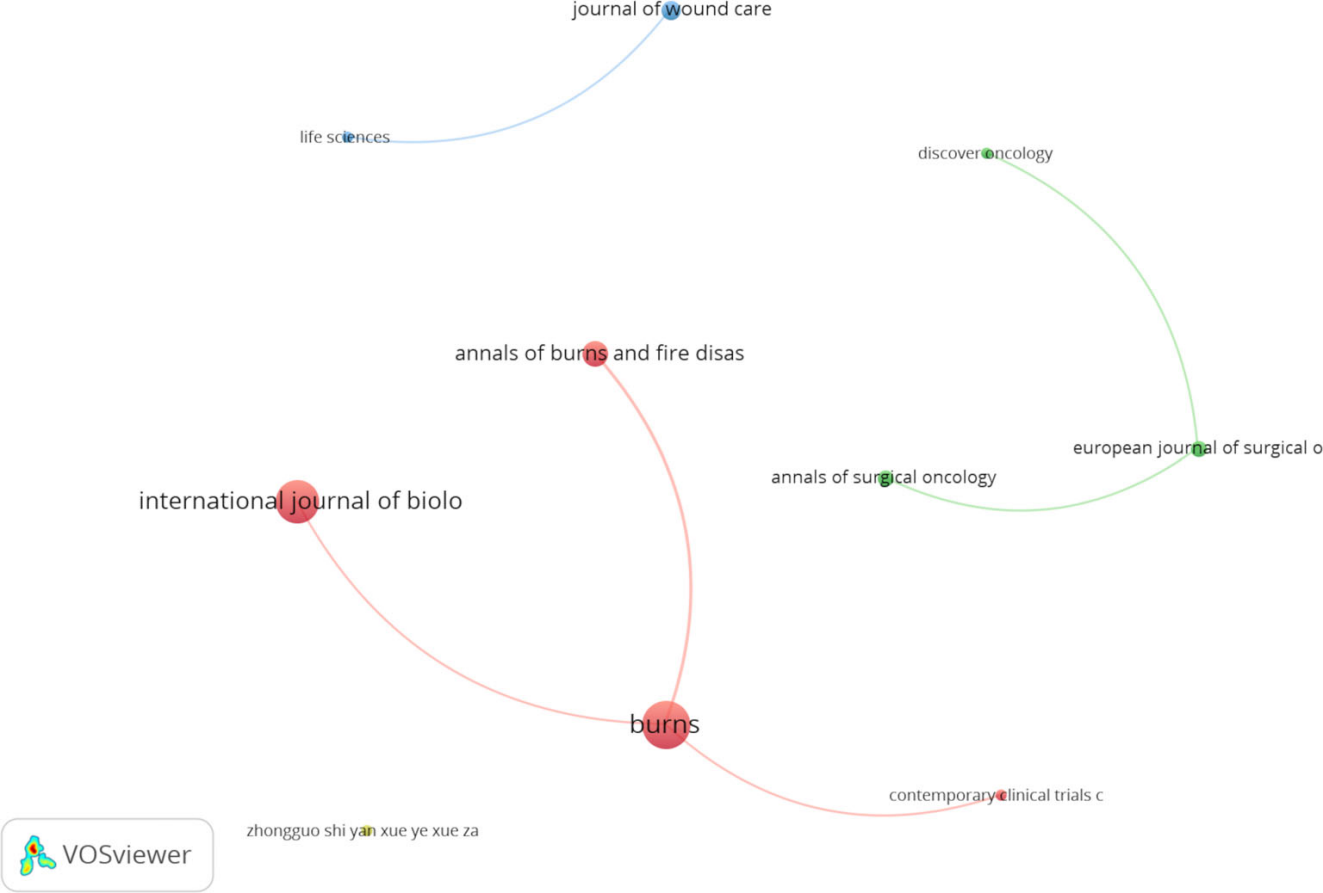
Visualization map for the 10 most cited journals in bromelain application research, according to bibliometric analysis in the database Scopus (2020–2024). Data collected on 20 September 2025. Analysis performed using VOSviewer, version 1.6.20.

This lack of interconnection suggests that the research topics conveyed by these journals are, to some extent, isolated, forming ‘islands of knowledge’ that do not communicate directly through citations. The different clusters (colors) reinforce this segmentation, showing that bromelain research is distributed across distinct sub‐areas, each with its own communication channels and reference bases.

The visualization network in Fig. 3 reveals that scientific authority in the field of bromelain is determined by citation density and connectivity rather than publication volume alone. A significant contrast emerges when cross‐referencing these data: although Table 2 identifies journals such as Foods and Molecules as the most productive in terms of article count (*n*), they do not appear as primary nodes in Fig. 3. This suggests that, although these journals serve as gateways for a high volume of manuscripts, the core of academic influence and reference remains concentrated in specialized journals such as *Burns* and the *International Journal of Biological Macromolecules*, for which the prominent nodes reflect a more robust and centralized technical impact within the network.

The connections and cluster formations in Fig. 3 reinforce this hierarchy of influence. The red cluster, led by the journal *Burns*, demonstrates superior link strength, integrating with high‐impact clinical and biochemical journals that, as shown in Table 2, maintain significant Impact Factors (IF > 4.0). This configuration indicates that the citation network prioritizes thematic depth and specialization; the thicker links between the nodes in the map illustrate where knowledge is effectively validated and reused, highlighting that bromelain science is consolidated through an elite axis connecting regenerative medicine to macromolecular biochemistry.

The *Burns* cluster is more related to biomedical applications, such as the use of bromelain as an option for surgical intervention for debridement in burn treatment.^23^ This fragmentation of the network reflects the multifaceted nature of bromelain research, which spans from food technology to biology and medicine.

The analysis of the institutions for bromelain research, shown in Table 3, presents the main funding sources driving the research. St George Hospital and Voronezhskiy Gosudarstvenniy Universitet stand out as the institutions with the highest number of publications, with 19 and 18, respectively. The presence of affiliations from different countries, such as UNSW Sydney (Australia) and the Università degli studi di Bari Aldo Moro (Italy), suggests that a diverse global network conducts the research.

**Table 3:** Ranking of affiliation with the highest number of publications in bromelain application research, according to bibliometric analysis in the Scopus database (2020–2024)

Ranking	Affiliation	*n*
1	University of New South Wales (UNSW)	28
2	St George Hospital	19
3	Voronezh State University	18
4	Sevastopol State University	14
5	Jiangnan University	12
6	Ben‐Gurion University of the Negev	12
7	Mucpharm Pty Ltd	11
8	Russian Academy of Sciences	11
9	University of Bari Aldo Moro	11
10	Russian Academy of Sciences	11

*Note: n*, number of publications. Source: data collected on 20 September 2025.

For the analysis of funding agencies (Table 4), the most emphasized aspect of the analysis is the dominance of Chinese funding agencies. The National Natural Science Foundation of China overwhelmingly leads the ranking with 51 citations, which corresponds to more than double the second‐place finisher, the National Key Research and Development Program of China (*n* = 23). This data suggests that China plays a dominant role in funding bromelain research. The inclusion of the National Council for Scientific and Technological Development (Conselho Nacional de Desenvolvimento Científico e Tecnológico – Brazil) in the top 3 indicates the relevance of Brazil in financing bromelain research, which may be related to its biodiversity and natural resources.

**Table 4:** Ranking of Funding Sponsor with the highest number of publications in bromelain application research, according to bibliometric analysis in the Scopus database (2020–2024)

Ranking	Funding sponsor	*n*
1	National Natural Science Foundation of China	51
2	National Key Research and Development Program of China	23
3	Conselho Nacional de Desenvolvimento Científico e Tecnológico	20
4	Coordenação de Aperfeiçoamento de Pessoal de Nível Superior	19
5	Council of Scientific and Industrial Research, India	16
6	Ministry of Science and Technology, Taiwan	12
7	Fundação de Amparo à Pesquisa do Estado de São Paulo	12
8	Russian Science Foundation	11
9	Science and Engineering Research Board	10
10	United Arab Emirates University	9

*Note: n*, number of publications. Source: data collected on 20 September 2025.

The analysis of the geographical distribution of scientific output, as shown in Table 5, presents the country's leading bromelain research. China emerges as the principal research hub, with a significantly superior volume of publications, totaling 150 publications and corresponding to a proportion of 0.1659 of the total sample. This prominence can be interpreted as a reflection of policies that invest in and incentivize research and development. India and Italy also demonstrate expressive output, occupying the second and third positions, respectively.

**Table 5:** Ranking of Countries with the highest number of publications in bromelain application research, according to bibliometric analysis in the Scopus database (2020–2024)

Ranking	Countries	*n*	%
1	China	150	16.59
2	India	105	11.62
3	Italy	75	8.30
4	Indonesia	68	7.52
5	United States	65	7.19
6	Malaysia	43	4.76
7	Iran	38	4.20
8	Germany	37	4.09
9	Brazil	36	3.98
10	Australia	30	3.32

*Note: n*, number of publications. Source: data collected on 20 September 2025.

The presence of nations from different continents in the ranking, such as Indonesia, USA and Brazil, highlights the global nature of bromelain research. The participation of Brazil, in particular, with 36 publications, reinforces the contribution of countries with rich biodiversity, given that bromelain is an enzyme extracted from the pineapple. These geographical data confirm that, although research is widely distributed, there is a clear concentration of production in nations with strong funding and research agendas.

Table 6 presents the ranking of the top 10 most cited articles on bromelain application research, according to a bibliometric analysis in the Scopus database (2020–2024). The table details the rank, title, authors, journal, number of citations, the journal's IF and CiteScore (CS), publication year and bibliographic reference for each article.

**Table 6:** Ranking of the 10 articles with the highest number of citations in bromelain application research, according to bibliometric analysis in the Scopus database (2020–2024)

Ranking	Title	Source	Citations	IF	CS	Year	Reference
1	Semiconducting Polymer Nanomaterials as Near‐Infrared Photoactivatable Protherapeutics for Cancer	Accounts of Chemical Research	365	22.38	36.3	2020	Li and Pu^21^
2	Effect of enzymatic hydrolysis on molecular weight distribution, techno‐functional properties and sensory perception of pea protein isolates	Innovative Food Science and Emerging Technologies	180	5.91	8.20	2020	Garcia Artega *et al*.^28^
3	Integral valorization of pineapple (*Ananas comosus* L.) By‐products through a green chemistry approach towards Added Value Ingredients	Foods	116	5.10	3.00	2020	Campos *et al*.^30^
4	Identification of antihypertensive peptides from mung bean protein hydrolysate and their effects in spontaneously hypertensive rats	Journal of Functional Foods	107	4.45	6.80	2020	Sonklin *et al*.^26^
5	Preparation of a moderate viscosity, high performance and adequately‐stabilized soy protein‐based adhesive via recombination of protein molecules	Journal of Cleaner Production	99	9.29	13.10	2020	Xu *et al*.^31^
6	Characterization of structure, physicochemical properties, and hypoglycemic activity of goat milk whey protein hydrolysate processed with different proteases	LTW	94	6.00	9.60	2022	Du *et al*.^27^
7	Alcalase and bromelain hydrolysis affected physicochemical and functional properties and biological activities of legume proteins	Food Structure	89	4.55	4.30	2021	Xu *et al*.^29^
8	Multifunctional bioactive peptides derived from quinoa protein hydrolysates: Inhibition of α‐glucosidase, dipeptidyl peptidase‐IV and angiotensin I converting enzymes	Journal of Cereal Science	80	3.61	5.10	2020	Mudgil *et al*.^32^
9	Antioxidant activity and peptidomic analysis of porcine liver hydrolysates using alcalase, bromelain, flavourzyme and papain enzymes	Food Research International	76	6.46	8.10	2020	López‐Pedrouso *et al*.^25^
10	Identification and characterization of cholesterol esterase and lipase inhibitory peptides from amaranth protein hydrolysates	Food Chemistry: X	74	6.44	5.40	2021	Fisayo Ajayi *et al*.^33^

*Note:* IF, Impact Factor of the corresponding year; CS, CiteScore of the corresponding year. Source: data collected on 20 September 2025.

The analysis of the most cited articles provides an overview of the areas that are driving bromelain research. The article by Li and Pu^24^ on nanomaterials for cancer stands out with 365 citations, suggesting a growing interest and impact of bromelain applications in cutting‐edge areas like nanomedicine. This finding is particularly relevant because it shows that the field is evolving from traditional applications toward innovative health solutions.

In addition to the cancer study, the table includes works on the antioxidant activity of protein‐derived peptides^25^ and the production of antihypertensive peptides,^26^ reinforcing bromelain's role in generating biopeptides with human health benefits.

The majority of articles explore bromelain for protein modification, such as hydrolysates of whey protein^27^ and legume proteins,^28^, ^29^ and the valorization of by‐products.^30^ Such studies demonstrate the enzyme's utility in improving the physicochemical and functional properties of food, in addition to its contribution to sustainability. The article by Xu *et al*.^31^ on protein‐based adhesives illustrates the multifaceted potential of bromelain in unconventional areas.

Figure 4 comprises a network visualization map, generated by bibliometric software, that illustrates the 50 most frequently cited keywords in bromelain application research. Each node (circle) on the map represents a keyword, and the proximity between the nodes indicates the frequency with which these terms co‐occur in the articles. The size of the node is proportional to the term's frequency, and the colors define thematic research clusters.

**Figure 4:**
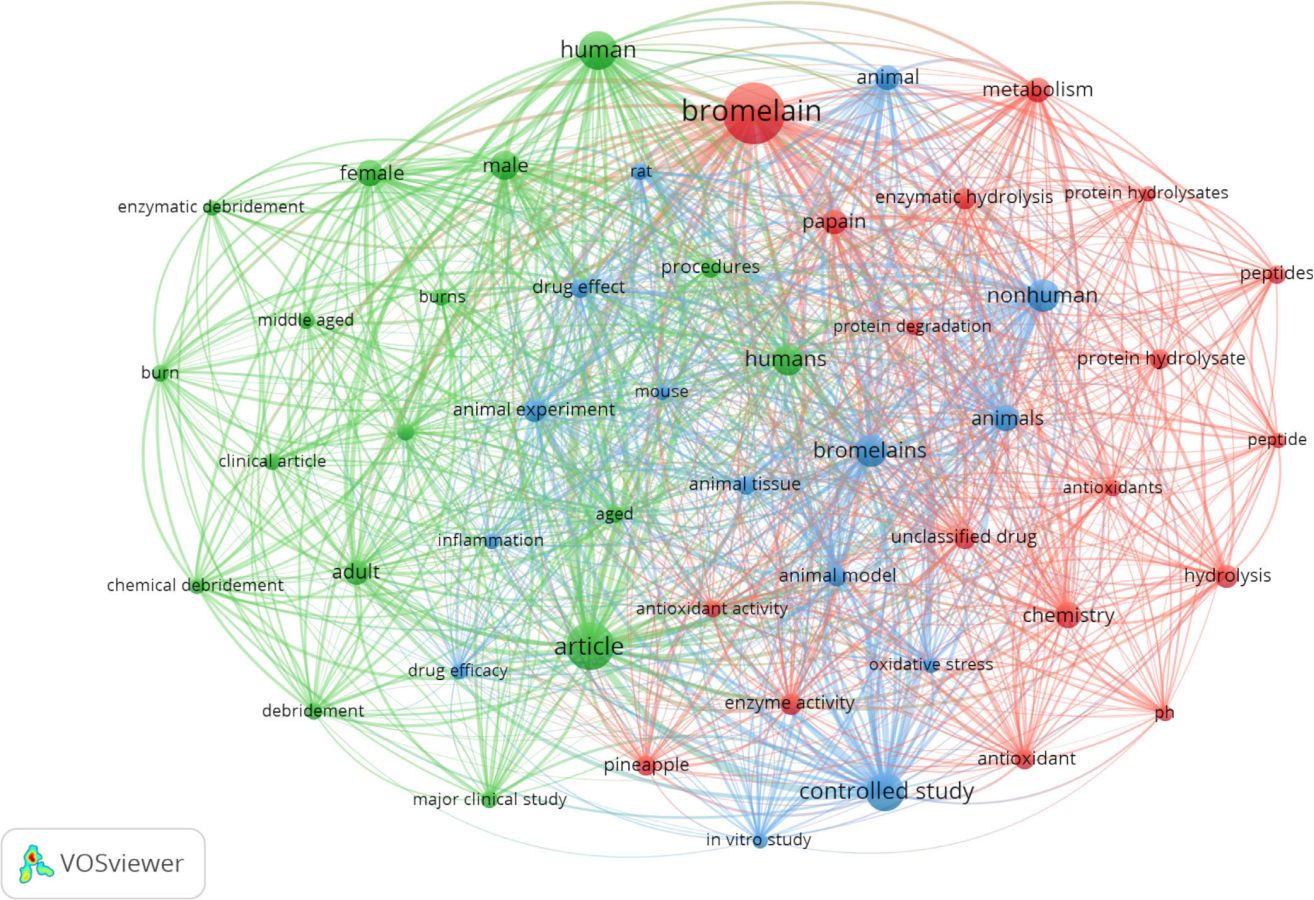
Visualization map for the 50 most cited keywords in bromelain application research, according to bibliometric analysis in the database Scopus (2020–2024). Data collected on 20 September 2025. Analysis performed using VOSviewer, version 1.6.20.

The visualization demonstrates that the field is not monolithic but is divided into three major research fronts. The green cluster, centered on terms such as ‘human’ and ‘clinical article’, indicates a strong and growing interest in the therapeutic and clinical applications of the enzyme, such as the treatment of inflammation. This aligns with the high impact of the article by Li and Pu.^24^ on nanomedicine, which directs research toward the healthcare sector.

The blue cluster, with terms such as ‘protein degradation’ and ‘food’, and the red cluster, with ‘peptides’ and ‘antioxidant’, represent the food applications and the investigations into biological properties. These clusters illustrate the continuing relevance of bromelain as an industrial enzyme and for the production of bioactive peptides, corroborating the discussion on articles that explore protein hydrolysis for nutritional and functional purposes.

The interconnection between these clusters, symbolized by the centrality of terms such as ‘bromelain’, illustrates the multifaceted nature of the research, where discoveries in one area, such as the antioxidant properties of peptides (red cluster), can inform and drive clinical applications (green cluster). The presence of ‘controlled study’ as a central term also suggests a methodological rigor in studies seeking to validate these applications.

The most representative keywords for each cluster, along with their occurrence values, are detailed in the Supporting information (Table S1).

### Industrial applications of bromelain

Bromelain exhibits remarkable proteolytic properties, as previously observed, which grants it great commercial value in a vast range of applications spanning several industries. Table 7 presents the ranking of the Fields of Study concerning bromelain applications, according to the Scopus database.

**Table 7:** Ranking of Subject area Sponsor with the highest number of publications in bromelain application research, according to bibliometric analysis in the Scopus database (2020–2024)

Ranking	Subject area	*n*
1	Agricultural and Biological Sciences	302
2	Medicine	251
3	Biochemistry, Genetics and Molecular Biology	191
4	Chemistry	162
5	Pharmacology, Toxicology and Pharmaceutics	120
6	Chemical Engineering	106
7	Materials Science	74
8	Immunology and Microbiology	72
9	Engineering	65
10	Environmental Science	51

*Note: n*, number of publications. Source: data collected on 20 September 2025.

The analysis of the distribution of publications by field of knowledge corroborates the thematic trends identified in Fig. 4. Bromelain research is predominantly rooted in biological and medical sciences, with ‘Agricultural and Biological Sciences’, ‘Medicine’ and ‘Biochemistry, Genetics and Molecular Biology’ accounting for the highest concentrations of articles. This distribution reflects the dual focus of bromelain: its origin in a biological source and its vast therapeutic and enzymatic potential.

Although the core of the research is biological, the diversity of knowledge areas in the table underscores the multifaceted nature of the field. The presence of publications in ‘Chemical Engineering’, ‘Materials Science’ and ‘Environmental Science’ demonstrates that research has expanded towards innovative solutions in non‐traditional sectors, such as the production of biomaterials and the development of sustainable processes. This suggests that bromelain is consolidating its position as a versatile biomaterial, capable of acting in a wide range of industrial and biotechnological applications.

The Agricultural and Biological Sciences field, with 302 publications in the analyzed period, proves to be the most prolific in bromelain research. Most studies in this area focus on obtaining functional peptides, particularly within sub‐disciplines such as food science, enzymology and protein hydrolysis, aimed at the development of bioactive compounds with health‐promoting properties.

Fisayo *et al*.^33^ evaluated the potential of amaranth protein hydrolysates as inhibitors of enzymes related to lipid metabolism. To this end, they generated hydrolysates using different enzymes and hydrolysis times, observing that those obtained with bromelain, especially the 4‐h hydrolysate, exhibited the greatest inhibitory effect on pancreatic lipase and cholesterol esterase. Peptide analysis identified bioactive sequences with the predicted capacity to inhibit these enzymes. The authors emphasize that this is the first work to report amaranth‐derived peptides with this potential, highlighting their application in the development of functional foods aimed at metabolic health.

To evaluate the potential of the King Boletus mushroom as a source of bioactive peptides obtained by enzymatic hydrolysis with bromelain, Kaprasob *et al*.^34^ fractionated the protein hydrolysate by ultrafiltration into different molecular sizes. The fraction with the lowest molecular weight (< 1 kDa) exhibited the highest hydrophobicity, the highest content of hydrophobic amino acids, and the most relevant biological activities. This fraction displayed strong antioxidant action and significant inhibition of the angiotensin I‐converting enzyme (ACE). Peptide characterization revealed new sequences with antioxidant capacity and antihypertensive effect, including the peptide LIYAQGFSK, which showed the highest binding energy to ACE. The study demonstrates that bromelain is effective in obtaining functional peptides from the King Boletus mushroom, reinforcing its potential as a tool to generate ingredients with antioxidant and antihypertensive properties.

Chandrasekaran *et al*.^35^ investigated chickpea proteins as a source of bioactive peptides with antidiabetic potential. For this, they compared two enzymatic treatments: a simulated digestive system (pepsin/pancreatin) and bromelain hydrolysis. The obtained peptides were characterized regarding their sequences, properties and molecular interactions with enzymes related to glucose metabolism. Molecular docking showed that some peptides inhibited dipeptidyl peptidase‐4 (DPP‐IV), in addition to interacting with bitter receptors, which may have beneficial effects in the management of type 2 diabetes. Although the hydrolysates obtained by pepsin/pancreatin exhibited greater affinity for DPP‐IV, the study highlights bromelain as a relevant alternative for generating functional peptides from chickpea, reinforcing its potential in formulating food ingredients aimed at diabetes prevention.

In the Medicine field, we have studies such as recommendations on the use of bromelain‐based enzymatic debridement (Nexobrid®; MediWound, Wilmington, DE, USA) in the treatment of burns, based on the practical experience of Italian specialists. Ranno *et al*.^36^ reached a consensus guided by the Delphi method, with the participation of burn centers in Italy that reported accumulated experience in over 1000 treated patients. At the end of the process, there was total agreement on virtually all clinical recommendations evaluated, resulting in a set of guidelines applicable to the use of Nexobrid® in burn surgery. The study emphasizes that these guidelines, although based on the Italian reality, reinforce the efficacy and safety of bromelain as a tool in enzymatic debridement and will serve as a basis for future updates as new evidence emerges.

Valle *et al*.^37^ evaluated the safety and preliminary efficacy of treating mucinous peritoneal tumors with BromAc®, a combination of bromelain (Brom) and acetylcysteine (Ac), known for its mucolytic activity. The clinical study involved 20 patients, in whom BromAc® was administered via a drainage tube directly into the tumor sites. Although most patients experienced mild to moderate adverse events, there were no deaths or anaphylactic reactions. An objective response to the treatment was observed in 73.2% of the treated sites, with significant mucin dissolution assessed radiologically. The results indicate that bromelain, in combination with acetylcysteine, has a manageable safety profile and therapeutic potential as a minimally invasive treatment for inoperable mucinous tumors.

The inclusion of the *Chemical Engineering* field, with over 100 publications, in the ranking demonstrates the interdisciplinary reach of bromelain research, extending beyond its biological uses. Mala and Anal^38^ developed hybrid hydrogel beads based on pectin and resistant starch to encapsulate bromelain, aiming to protect its activity during gastrointestinal passage and thermal processing. The study evaluated different pectin‐to‐resistant starch ratios, observing that the 4.5:1.5 (w/w) combination provided the highest encapsulation efficiency, improved gastric stability, and sustained release of bromelain. The encapsulated enzyme maintained high activity even after thermal treatments up to 95 °C and was released more rapidly in simulated intestinal fluid (pH 7.4) than in gastric fluid (pH 1.2). The results indicate that hydrogel encapsulation is an effective strategy to preserve bromelain's functionality in food and nutraceutical applications.

#### Structural and functional aspects of bromelain

Bromelain is a complex mixture of cysteine proteases belonging to the Bromeliaceae family, characterized by the presence of an active site composed primarily of cysteine and histidine residues. The catalytic mechanism of bromelain is based on nucleophilic attack mediated by the thiol group of cysteine, which enables the cleavage of peptide bonds in protein substrates. This structural configuration is directly associated with its high proteolytic efficiency and broad substrate specificity.^39^

In addition to its catalytic residues, bromelain presents a three‐dimensional conformation stabilized by disulfide bonds, which play a crucial role in maintaining enzyme stability under specific pH and temperature conditions. However, this structure is also sensitive to environmental factors such as heat, extreme pH and oxidative conditions, which may lead to denaturation and loss of activity.^7^

The relationship between structure and function is fundamental to understanding bromelain applications. Its ability to hydrolyze structural proteins such as collagen and keratin underlies its use in meat tenderization, enzymatic debridement and cosmetic exfoliation. At the same time, its sensitivity to processing conditions highlights the importance of stabilization strategies, such as immobilization and microencapsulation.^40^

#### Food industry

According to the US Food and Drug Administration,^41^ bromelain is classified as a food additive, being employed as a flavor enhancer, flavoring agent or adjuvant, processing aid, and texturizing agent. This proteolytic enzyme exhibits broad applicability in the food industry, especially in meat tenderization, beverage clarification and the production of protein hydrolysates.^42^ Some of the main applications of bromelain are illustrated in Fig. 5.

**Figure 5:**
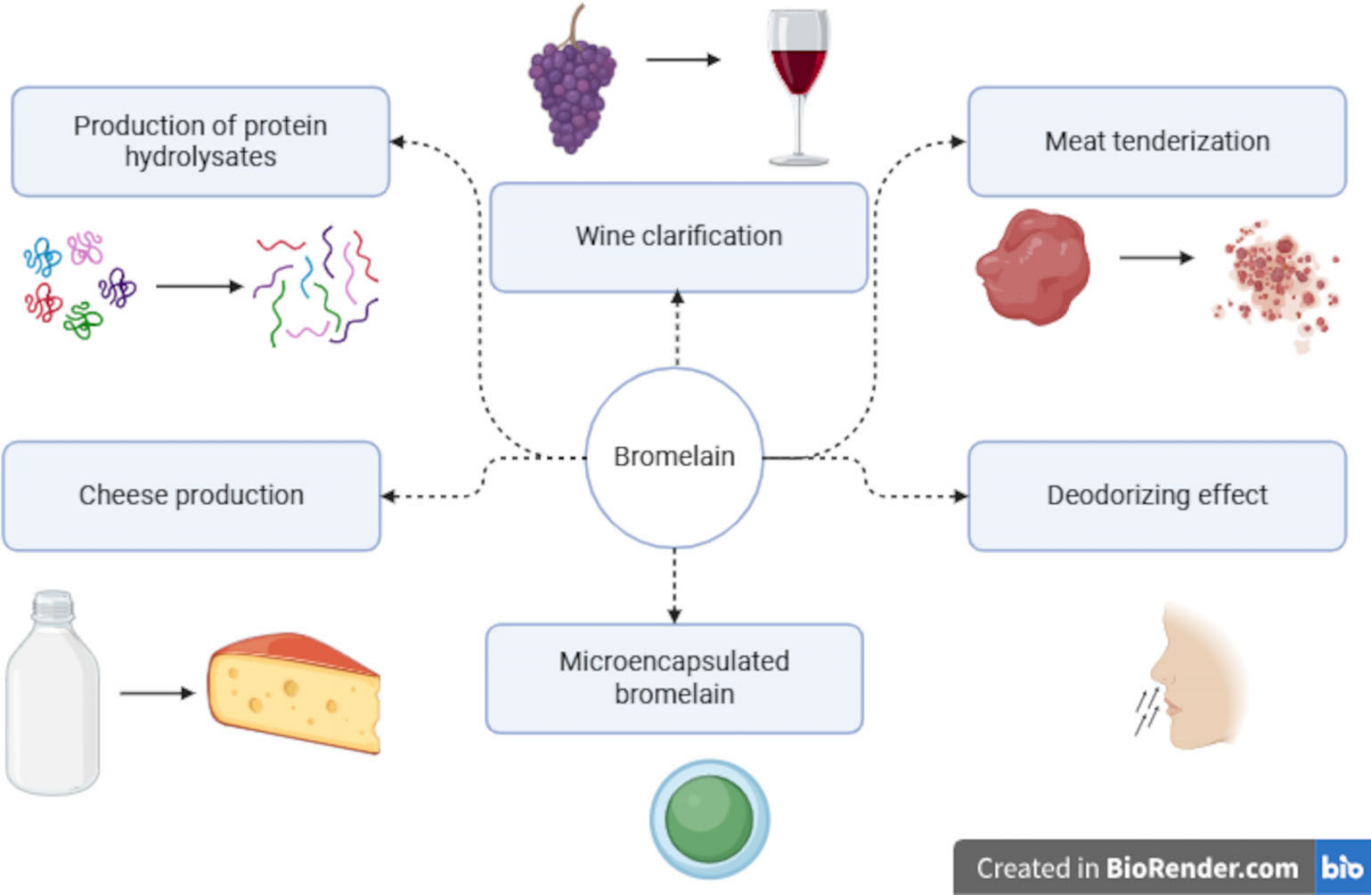
Applications of bromelain in the food industry.

For the production of protein hydrolysates, Nurdiani *et al*.^43^ utilized by‐products from *Pangasius* sp. and found high concentrations of glycine, l‐glutamic acid and l‐aspartic acid in the amino acid profiles of the hydrolysates. In a similar study, Noman *et al*.^44^ obtained protein hydrolysates from hybrid sturgeon (*Huso dauricus* × *Acipenser schrenckii*) using bromelain, identifying peptides with antioxidant activity, which demonstrate potential for application in the food industry as natural substitutes for synthetic antioxidants.

Selamassakul *et al*.^45^ utilized bioactive peptides from rice protein hydrolyzed by bromelain and observed that the hydrolysates have the potential to be used as a functional additive in nutraceutical food and beverage products, promoting consumer health at the same time as formulating products with pleasant sensory characteristics.

Begum *et al*.^46^ emphasize that flavor is one of the main factors determining food quality, directly influencing consumer preferences and purchase intent. Aiming to reduce undesirable odors and enhance flavor, Qin *et al*.^47^ applied bromelain, papain and a mixture of both enzymes in saline solutions to treat pig trotters. The results demonstrated that the combined bromelain and papain treatment significantly reduced the aldehydes nonanal and octanal, which are compounds associated with undesirable odors and off‐flavors. Qin *et al*.^47^ observed an increase in the concentration of esters such as ethyl propionate, ethyl isobutyrate, ethyl isovalerate, ethyl acetate, methyl acetate, and butyl acetate, which contribute to a more pleasant flavor profile. In addition to the production of protein hydrolysates and its deodorizing and flavoring effect, one of the main applications of bromelain is in the meat and fish industry, aiming to improve product tenderization and texture.

Mohd Azmi *et al*.^6^ evaluated recent trends and future perspectives for the application of plant proteases in meat tenderization and observed that studies in this area have gained popularity as a result of the positive changes in the sensory characteristics of the meat, the presence of bioactive compounds that possess antimicrobial and antioxidant properties, and lower production costs.

Zhou *et al*.^48^ tested the effect of ultrasound combined with enzymatic treatment on the tenderization of dried shrimp and concluded that this method can be used for softening during production, as it is a simple and effective process as a result of the breakdown of the shrimp's myofibrillar proteins. With the aim of obtaining a soft pork paste, Saengsuk *et al*.^49^ conducted a study preparing pastes with different levels of bromelain (0 and 5 g kg^−1^ w/w), kappa‐carrageenan (5 and 10 g kg^−1^w/w) and rice‐to‐mung bean ratios (0:0, 1:0, 0:1 and 0.5:0.5). It was concluded that bromelain has the potential to produce a softer meat paste.

In the beverage industry, bromelain can be used for beverage clarification, as demonstrated by Benucci *et al*.,^50^ who observed the effect of free and immobilized stem bromelain on the protein turbidity of white wine. As a result, Benucci *et al*.^50^ concluded that 10 g L^−1^ of bromelain immobilized in chitosan beads is required after 24 h of treatment to reduce wine turbidity by 70%, using a laboratory‐scale stirred reactor.

Khan *et al*.^51^ utilized various plant extracts, including bromelain, in cheese production. The results demonstrated that such extracts can be better and more economical commercial sources, acting as substitutes for animal and microbial rennet. In cheese production, Komansilan *et al*.^52^ used bromelain extract as an alternative rennet substitute. The results indicated that a concentration of 30 g kg^−1^ of the extract produced cottage cheese with the best physicochemical characteristics, demonstrating that the crude enzyme can be a viable and low‐cost replacement in cheesemaking.

Vergara Alvarez *et al*.^9^ utilized dried bromelain extract in raw and pasteurized milk to produce fresh cheese. The results revealed that, regardless of the type of milk (raw or pasteurized), the product with the highest sensory acceptance was obtained using 50 g kg^−1^ of the dried extract, concluding that the use of bromelain shows potential as a plant‐based coagulant.

Aiming to preserve the enzymatic activity of bromelain, Tang *et al*.^53^ performed microencapsulation via complex coacervation (CoCo). It was reported that the enzyme's proteolytic activity was fully recovered from the bromelain–alginate coacervation powder, indicating the potential for scalable and cost‐effective industrial application.

This preservation effect can be explained by the formation of a protective polymeric matrix surrounding the enzyme, which acts as a physical barrier against external stressors such as heat, oxygen, and pH variations.^54^ During thermal processing, this encapsulating layer reduces protein denaturation by limiting direct exposure to high temperatures and restricting molecular mobility, thereby maintaining the structural integrity of the enzyme. Additionally, the controlled release of bromelain from the coacervate system may further contribute to the retention of its activity under processing conditions.

#### Pharmaceutical industry

Bromelain exhibits therapeutic effects exerted by a multifaceted mechanism, which includes anti‐inflammatory, analgesic, antiangiogenic and antioxidant properties. These properties make it useful in the pharmaceutical industry for the treatment of inflammation and the combat against oxidative stress.^55^

Ferah Okkay *et al*.^56^ observed that the enzyme exhibited dose‐dependent protective activity against ocular toxicity induced by cisplatin by regulating oxidative stress, suggesting that the enzyme may be an adjuvant capable of protecting the eyes from cisplatin‐induced toxicity.

When evaluating the nephroprotective effect of bromelain, El Demerdash *et al*.^57^ tested the enzyme against oxidative stress stimulated by aluminum chloride in rats. It was concluded that the enzyme possesses a significant protective effect and managed to neutralize the toxic influence of aluminum chloride, indicating an innovative approach in the therapy for metal toxicity, as a result of its antioxidant and chelating properties.

*In vivo* and *in vitro* studies reported by Sharma and Vimal^58^ demonstrate other biological activities of bromelain, including fibrinolytic (dissolution of blood clots), antithrombotic (inhibition of thrombus formation or growth) and anti‐edematous (reduction of edema, i.e. fluid accumulation in tissues) activities. Sharma and Vimal^58^ also suggest that the enzyme has therapeutic potential for the treatment of conditions such as bronchitis, surgical trauma and sinusitis.

Kumar *et al*.^59^ conducted a pharmacological evaluation of bromelain in Swiss albino mice with induced Alzheimer's disease. The results demonstrated that bromelain, both alone and in combination with donepezil, was able to prevent spatial learning and memory deficits induced by aluminum chloride and d‐galactose, in addition to attenuating cognitive impairment in the cerebral cortex and hippocampus. The findings indicate neuroprotective potential, with attenuation of oxidative damage, neuroinflammation and Aβ1‐42. aggregation.

#### Other industries

In addition to the food and pharmaceutical industries, bromelain has also been applied in other sectors. In addition to the food and pharmaceutical industries, bromelain has also been applied in other sectors. In the cosmetic industry, bromelain has been increasingly explored as a functional bioactive ingredient as a result of its proteolytic activity and compatibility with skin physiology. Its primary mechanism involves the hydrolysis of keratin proteins in the stratum corneum, enabling the controlled removal of corneocytes and promoting skin renewal. This enzymatic exfoliation process results in improved skin texture, enhanced radiance and increased permeability to active compounds, making bromelain particularly valuable in cosmeceutical formulations. Compared to traditional mechanical and chemical exfoliants, enzymatic systems based on bromelain offer a milder and more selective action, reducing the risk of irritation and preserving the integrity of the skin barrier. This characteristic makes bromelain especially suitable for sensitive and acne‐prone skin, where aggressive exfoliation methods may lead to inflammation or barrier disruption.^60^

Bromelain has been incorporated into a wide range of cosmetic products, including enzymatic peels, facial masks, cleansers and anti‐aging formulations. In these applications, it acts not only as an exfoliating agent, but also as a multifunctional ingredient with anti‐inflammatory and skin‐conditioning properties. The concept of enzymatic cosmetics (‘enzymocosmetics’) has emerged as a promising approach in modern skincare, emphasizing the use of proteolytic enzymes such as bromelain and papain to achieve effective yet gentle skin renewal.^61^

Studies evaluating bromelain‐based treatments on skin tissues have demonstrated its ability to selectively degrade proteinaceous structures without causing significant damage to surrounding viable cells, highlighting its potential as a safe and controlled agent for dermatological and cosmetic applications.^62^

In line with this mechanism, bromelain has been reported to exhibit dermocalming properties, promoting a soothing effect on skin subjected to post‐depilatory or other aggressive treatments.^63^ Additionally, it has been employed in aesthetic procedures, often in combination with papain, to reduce the thickness of the stratum corneum and improve skin appearance through enhanced cell renewal.

Beyond topical skin applications, bromelain has also been incorporated into oral care formulations. Patil *et al*.^64^ compared the efficacy of extrinsic stain removal using toothpastes containing abrasive components (perlite and calcium carbonate) and enzymatic agents (papain and bromelain), demonstrating that enzymatic formulations were equally effective. Notably, although abrasive toothpastes require careful use because of potential enamel wear, enzymatic formulations showed no contraindications under the evaluated conditions.

In the textile industry, bromelain has been utilized to improve the quality of proteolytic dyeing processes.^65^, ^66^ In this context, it has been increasingly explored as an effective proteolytic agent for the processing of protein‐based fibers such as silk and wool.

In silk processing, bromelain has demonstrated high efficiency in sericin removal during degumming, at the same time as preserving fibroin integrity, resulting in improved mechanical properties and enhanced fabric softness.^67^ Comparative studies have further shown that enzymatic treatments using bromelain cause less structural damage than conventional chemical methods, highlighting their potential as environmentally friendly alternatives.^68^

Similarly, in wool processing, bromelain and other proteolytic enzymes act on the keratin structure of fiber surfaces, modifying cuticle layers and enhancing dye uptake and color uniformity. These enzymatic treatments have been associated with improved color fastness, increased softness and better overall fabric performance.^69^ Moreover, bromelain‐based processes have been proposed as sustainable alternatives to traditional chlorination methods, significantly reducing environmental impact and chemical consumption in textile finishing operations.^70^

#### Challenges and perspectives

The diversity of bromelain sources and their distinct structural and biochemical characteristics directly influence enzyme performance and pose specific challenges during extraction, purification and industrial application.

Bromelain is classified as a cysteine protease, such that its active site contains the amino acid cysteine conjugated with histidine. This type of enzyme is generally active at a neutral pH but can exhibit some activity under acidic conditions. Bromelain has optimal activity at a pH range of 6.0 to 8.0, but it rapidly loses its activity at temperatures above 70 °C.^71^

According to Ramli *et al*.^72^ bromelain can be extracted from various parts of the pineapple (*Ananas comosus*), including the stem, the fruit (or pulp), the flowers and the peel. However, only the stem and the fruit yield commercially relevant quantities of the enzyme, with the stem being the primary source for industrial extraction as a result of its higher bromelain concentration and lower cost because it is a by‐product of harvesting.

The proteases found in the pineapple stem are denominated stem bromelain, possessing the systematic number EC 3.4.22.4, whereas those extracted from the fruit are called fruit bromelain, following the numbering EC 3.4.22.5.^1^ Therefore, to carry out industrial processes, it is necessary to understand the optimal conditions of the enzyme, which can vary according to the location of extraction. Stem bromelain is considered relatively stable, maintaining activity between 40 and 60 °C, a range in which several enzymes are denatured.^73^ As a result of the processes used for extraction and purification, commercial bromelain has become a high‐cost product.^74^

Bromelain is defined as a complex of proteases from the Bromeliaceaea family, found in different parts of the plant, including the stem, leaf, root and fruit. Although the family encompasses approximately 3652 species distributed across 78 genera,^75^ few species are explored, resulting in a scarce exploration of bromelain derived from other representatives.

Silva and Tambourgi^76^ extracted and evaluated the stability of bromelain from the leaves of purple curauá (*Ananas erectifolius*), which showed best stability at pH 8 at 10 °C, whereas Silva *et al*.^77^ using bromelain extracted from the leaves of macambira (*Bromelia laciniosa*), found its optimum points at pH 7 at 47 °C. Salese *et al*.^78^ used the fruits of *Bromelia serra* to prepare a crude extract with proteolytic activity, which showed an optimal pH range of 6.03–9.05, managing to retain 80% at temperatures of 23, 37 and 45 °C (120 min), and becoming inactive in 10 min at 75 °C.

Such results demonstrate significant differences in the bromelain types from different species of the Bromeliaceaea family, indicating a growing trend to explore alternative sources of this enzyme, aiming to diversify the means of extraction and increase productive efficiency.

Regarding industrial applications, the diversification of bromelain sources opens new possibilities for the development of products with innovative applications. The food industry, for example, can benefit from proteases adapted to different substrates and processing conditions, whereas the pharmaceutical industry can explore the anti‐inflammatory, antitumoral and immunomodulatory properties of enzymes derived from different sources. Furthermore, there is a growing interest in using bromelain in cosmetic products and biomaterials, reinforcing the versatility of this enzyme class.

## CONCLUSIONS

Bromelain is an enzyme of great relevance and versatility, with applications that extend from the food and pharmaceutical industries to emerging sectors such as biomaterials and cosmetics, solidifying its role as a multifunctional bioactive agent.

The bibliometric analysis revealed that, although research is global, it is heavily concentrated in nations with significant investment in science and technology, particularly China. The fragmentation into sub‐areas, evidenced by the journal and keyword clusters, reflects the multifaceted nature of the enzyme, with different fields exploring its distinct properties and potential.

Considering the bibliometric mapping conducted in this study, the exploration of new sources from the Bromeliaceaea family, beyond the traditional pineapple, emerges as a promising strategy to overcome challenges such as thermal instability, thereby expanding the possibilities for developing more efficient and innovative products.

Despite these advances, some important areas within food science remain underexplored. In particular, limited attention has been given to the application of bromelain in complex food matrices subjected to industrial thermal processing, the long‐term stability of the enzyme under real processing and storage conditions, and its interactions with other food components such as proteins, lipids and polysaccharides. Additionally, the scalability of stabilization strategies, such as microencapsulation, and their economic feasibility in industrial applications still require further investigation.

Therefore, future research should prioritize these aspects to enable a more effective translation of bromelain from laboratory‐scale studies to practical and sustainable applications in the food industry. These findings reinforce the relevance of bromelain as the enzyme of the future, aligning with the emerging trends in its global application.

## CONFLICTS OF INTEREST

The authors declare that they have no conflicts of interest.

## AUTHOR CONTRIBUTIONS

GJdSN was responsible for conceptualization. GJdSN and TCCL were responsible for methodology. GJdSN, TCCL, GTSP, RdNA and ARdS were responsible for investigations. GJdSN and GTSP were responsible for data curation. GJdSN was responsible for formal analysis. GJdSN was responsible for writing the original draft. TCCL and MTC were responsible for reviewing and editing. MTC, RdNA, ArdS and MCG were responsible for supervision. MTC was responsible for validation. RdNA, ArdS and MCG were responsible for project administration.

## Supporting information


**Table S1.** Most frequent keywords for each cluster derived from the co‐occurrence analysis, including their occurrence values, according to bibliometric analysis in the Scopus database (2020–2024).

## Data Availability

All data generated or analyzed during this study are included in this published article.
