# Supplementary material for: Bromelain as the enzyme of the future: A global bibliometric mapping of its applications and emerging trends
Source: J Sci Food Agric. 2026 May 28;107(1):78–92. doi: 10.1002/jsfa.70751 (PMC13756560; doi:10.1002/jsfa.70751)
Supplement: Supplementary file 1 [file JSFA-107-78-s001.docx]

Supplementary Table S1. Most frequent keywords for each cluster derived from the co-occurrence analysis, including their occurrence values, according to bibliometric analysis in the Scopus database (2020-2024).

| **Cluster** | **Research Focus** | **Top Keywords (Occurrences)** |
| --- | --- | --- |
| Red | Enzymatic and biochemical characterization | Bromelain (636), chemistry (116), metabolism (106), papain (103) and hydrolysis (90). |
| Green | Clinical and human studies | Article (382), human (257), humans (168), male (154) and female (115). |
| Blue | Experimental and preclinical studies | Controlled study (253), bromelains (189), nonhuman (184), animals (117) and animal (113). |

**Source:** Data collected on September 20, 2025. Analysis performed using VOSviewer 1.6.20
